# Sweet Relief: Determining the Antimicrobial Activity of Medical Grade Honey Against Vaginal Isolates of *Candida albicans*

**DOI:** 10.3390/jof5030085

**Published:** 2019-09-09

**Authors:** Renée Hermanns, Niels A.J. Cremers, John P. Leeming, Esther T. van der Werf

**Affiliations:** 1 Triticum Exploitatie B.V., Sleperweg 44, 6222NK Maastricht, The Netherlands; renee@mesitran.com (R.H.);; 2 Infection Sciences Department, Severn Pathology, Southmead Hospital, Bristol BS10 5NB, UK; john.leeming@nbt.nhs.uk; 3 Bristol Medical School, University of Bristol, Canynge Hall, 39 Whatley Road, Bristol BS8 2PS, UK; esther.vanderwerf@bristol.ac.uk; 4 School of Medicine1, Taylor’s University, Jalan Taylor’s, 47500 Subang Jaya, Selangor D.E., Malaysia

**Keywords:** vaginitis, medical grade honey, candidiasis, *candida*, antibiotic resistance, recurrent vulvovaginal candidiasis

## Abstract

Recurrent vulvovaginal candidiasis (RVVC) is predicted to increase to almost 158 million cases annually by 2030. Extensive self-diagnosis and easily accessible over-the-counter (OTC) fungistatic drugs contribute to antifungal-resistance, illustrating the need for novel therapies. Honey possesses multiple antimicrobial mechanisms, and there is no antimicrobial resistance towards honey reported. We evaluated the susceptibility of five clinical isolates of *Candida albicans* and a control strain to regular honey and a medical grade honey (MGH) gel formulation (L-Mesitran, containing 40% honey and vitamins C and E) using an adapted version of the EUCAST protocol at pH 5.2, 4.6, and 4.0. 40% regular honey did not kill or inhibit *C. albicans*. In contrast, the minimal inhibitory concentration (MIC) of L-Mesitran was 25%–50%, while fungicidal effects occurred at a 50% concentration (MBC) of the MGH formulation, except for one strain which was not killed at pH 4.0. Overall, pH had little effect on antimicrobial activity. MGH formulation L-Mesitran has antimicrobial activity against *C. albicans* over a relevant pH range. The vitamin supplements or other components of L-Mesitran may enhance the antifungal activity of the honey. This study supports performing clinical trials for conditions, such as RVVC, to find an alternative to available OTC fungistatic drugs.

## 1. Introduction

*Candida albicans* causes 80–92% of all vulvovaginal candidiasis (VVC) [1,2]. The number of women worldwide suffering from recurrent VVC (RVVC) is expected to increase to almost 158 million by 2030 [3]. Routine microscopy and culturing are considered the standard to diagnose vaginitis in symptomatic women, but unfortunately these tests are often not done in practice, resulting in inappropriate medication and persistence of symptoms [4]. Self-diagnosis was found to have an accuracy rate of only 28% for *C. albicans* infection in self-treating women, and over-the-counter (OTC) antifungal agents are often ineffective [5,6,7]. Although vaginal isolates of *C. albicans* are largely azole sensitive currently, there is an increase in azole-resistance [8,9]. Possible causes of relapse include factors such as host immune status and sensitivity to fungal antigen, in addition to errors in self-diagnosis and self-medication. In this context, the availability of effective alternatives to azole antifungal agents with different mechanisms of action could have a significant impact on the global burden of health-care costs and patient’s lives. 

Honey is a natural substance that has been used for wound healing since ancient times [10]. The antibacterial and antifungal properties of honey can be ascribed to multiple mechanisms [11,12,13,14,15,16,17,18]. Honey contains over 200 different components, of which sugars, such as glucose and fructose, encompasses the main part (70%) [19]. These sugars have a strong osmotic activity, which leads to dehydration of the microorganisms. Glucose in contact with glucose oxidase releases small amounts of hydrogen peroxide that can kill the already damaged pathogens. The low pH makes it hard for pathogens to persist. In addition, honey contains several phytochemicals, including flavonoids and alkaloids that have antioxidative and direct antimicrobial effects, such as propolis, pinocembrin, chrysin, pinobanksin, galangin, quercetin, luteolin, and kaempferol [18,20,21]. Different honeys have different flavonoid profiles and thus antimicrobial activity, depending on the floral source of the nectar, geographic origin, environmental factors, and storage conditions [19]. Since the antimicrobial mechanisms of honey act via multiple mechanisms, it is hard for pathogens to develop resistance towards honey and this has not been described yet [22,23]. 

The MGH formulation investigated in this study (L-Mesitran) is indicated for fungating wounds. However, we received feedback from women using L-Mesitran successfully off-label for the treatment of vaginitis. To prevent off-label use and possibly expand its intended use for vaginitis in the future, we here wanted to find in vitro evidence that L-Mesitran is able to kill *C. albicans* and to justify further clinical studies. In this study, the susceptibility of *C. albicans* to honey and an MGH formulation (L-Mesitran) was tested by evaluating five isolates from vaginal samples of symptomatic patients. Tests were performed at a range of pH levels reflecting the natural acidic environment in the vagina. In contrast to bacterial vaginosis, a low pH is maintained during *Candida* infections [24]. 

## 2. Materials and Methods

*Candida* isolates were obtained from the Southmead Hospital in Bristol, UK. To ensure that the isolates were representative for the general population with common vaginal infections caused by *Candida*, we randomly selected five clinical *Candida albicans* isolates from vaginal swab specimens submitted for routine diagnostic purposes because of vaginal discharge. The isolates were confirmed to be *Candida albicans* strains by mass spectrometry (Biotyper MALDI-TOF; Bruker UK, Coventry). Samples were coded to prevent traceability back to the patient and guarantee anonymity. From two of these isolates a resistance profile was made, showing these strains would be susceptible to common antifungal agents, including azoles, amphotericin B, and caspofungin. We also included a control strain (NCPF3179) in our experiments which is extensively characterized and used in antimicrobial testing. These samples were not recruited as part of a specific study and patient consent was not sought. The five isolates and the control were tested against unprocessed Mexican Yucatan honey with hydrogen peroxide activity and L-Mesitran Soft (Triticum B.V., Maastricht, The Netherlands), a formulation containing 40% of the same Mexican Yucatan honey, hypoallergenic lanolin (Medilan™, Croda Inc.), propylene glycol, PEG4000, and vitamins C and E.

### 2.1. Preparation of Test Compounds and Microtiter Plates

Determination of the minimum inhibitory concentration (MIC) was performed based on the ‘EUCAST antifungal MIC method for yeasts’ but adapted to suit the nature and potency of the compounds under evaluation [25]. Yeast-nitrogen base (YNB, with amino acids; Sigma Y1250) was prepared at ×10 concentration according to manufacturer’s instructions and subsequently diluted to double strength (ds) concentration with sterile water before pH was adjusted to 5.2, 4.6 or 4.0 (+/- 0.05) with hydrochloric acid and filter sterilised. The rationale behind these pH values is that the vaginal pH normally lays between pH 3.8 and 4.2, while an imbalance in the vaginal ecosystem is often considered above pH 4.5, [26]. Honey products subjected to testing were provided by Theo Manufacturing B.V., Maastricht, The Netherlands (see Table 1). 

Maximum concentrations of MGH formulation L-Mesitran and pure honey in test wells were obtained by preparing emulsions in YNB rather than water according to:

x mL L-Mesitran + x mL ds YNB (resultant suspension equivalent to 20% honey),(1)

y mL honey + 0.25y mL H_2_O + 1.25y mL ds YNB (resultant suspension equivalent to 40% honey), (2)

where the resultant suspension was equivalent to 20% honey and 40% honey, respectively.

Each compound was suspended in ds YNB at pH 5.2, 4.6 and 4.0. To produce homogenous suspensions, they were heated to 37 °C and mixed vigorously using a vortex mixer. The suspensions were then serially diluted in two-fold steps in a 96-well plate (Corning® Costar®) using ×1 YNB of the appropriate pH as the diluent. The final well containing ×1 YNB and no test compound served as the control. 

### 2.2. Inoculum Preparation

*Candida* albicans cells were collected and suspended in sterile water at a density equivalent to a 0.5 McFarland standard as per EUCAST instructions: colonies from 18–24-h cultures on Sabouraud’s agar were suspended in 3 mL water and vortexed for 15 s. Turbidity was confirmed using a Vitek Densichek (BioMerieux). Cell density was confirmed to be within the acceptable range (1.0–5.0 × 10^6^ colony forming units per mL) by performing viable counts on Sabouraud’s agar. 

To minimise any additional dilution of the product in the final assay, 10 µL of 0.5 McFarland standard suspension was added to each 190 µL test well, rather than 100uL of a further 1/10 dilution to 100 µL as mentioned in EUCAST recommendations, resulting in the specified 0.5–2.0 × 10^5^ colony forming units per mL final concentration in test wells. From each control well, 10 µL samples were taken and plated onto Columbia Blood agar plates for purity checks and to serve as the pre-incubation comparator for post incubation cultures (see below). The comparator plates were incubated at 37 °C for 24 (+/-1) h. 

### 2.3. Plate Reading

Following 24-h incubation at 37 °C, microtiter plates were removed and 10 µL samples were taken from each test well, plated onto Columbia Blood agar and incubated at 37 °C for 24 h. Visual examination normally specified for reading microdilution assays was not useful because the test compounds rendered the wells turbid at the high concentrations tested. Hence, the minimum fungicidal concentration (MFC) was defined as the minimum dilution of test compound resulting in no growth of *C. albicans* in post-incubation subcultures (equivalent to a >10**^3^** kill). Likewise, the minimum inhibitory concentration (MIC) was defined as the minimum dilution of a test compound that resulted in similar or reduced growth of *C. albicans* in post-incubation subcultures when compared to pre-incubation controls. 

## 3. Results

**Figure 1:**
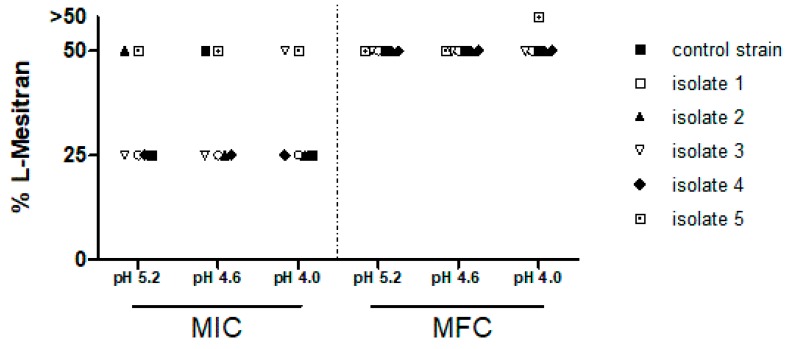
Activity of L-Mesitran against clinical *Candida* albicans isolates tested at pH 5.2, 4.6, and 4.0, shown as the minimum inhibitory concentrations (MIC) and minimum fungicidal concentrations (MFC). NCPF 3179 was added as the control strain.

The antifungal activities of both test compounds were consistent for all strains across the range of pH levels tested. The MICs for L-Mesitran remained in the dilution range of 25 to 50%, which is equivalent to a honey content of 10% and 20%, respectively. In almost all cases, the MFC for L-Mesitran was 50%, equivalent to 20 honey content (see Figure 1). In contrast, when testing the pure honey compound dilutions, no discernible inhibitory or cidal antimicrobial activity was noted at the highest concentration of 40% (*v*/*v*) honey tested, giving MIC and MFC values >40% honey for all strains at all pH levels tested. 

Minor strain-specific sensitivities to L-Mesitran were observed. An MIC of 50% L-Mesitran (20% (*v*/*v*) honey content) was needed for isolate 5 at every pH, while the MIC for remaining isolates and/or control strain typically was a 25% dilution of L-Mesitran (10% (*v*/*v*) honey content). Exceptions were isolate 2, the control strain, and isolate 3 at pH 5.2, pH 4.6, and pH 4.0, respectively, which also needed 50% L-Mesitran to effectively inhibit the growth. 

The MFC for *Candida* isolates 1 through 4, as well as the control strain, was 50% L-Mesitran in every pH condition, which has a honey content of 20% (*v*/*v*). At pH 4.0, 50% L-Mesitran, containing 20% honey, was not cidal for isolate 5.

## 4. Discussion

Current research suggests that MGH might offer a valuable therapeutic alternative in the treatment of VVC [14,15,18,27,28]. The results of this study showed no effect of 40% Mexican Yucatan MGH alone against *Candida albicans* isolates, whereas the MGH product had fungistatic and fungicidal activity. In vitro studies report relatively high minimal concentrations for the inhibition of *Candida* spp.; a study on the antimicrobial capacities of Slovenian honeys reported no inhibition of different *Candida* spp. at 50% (*v*/*v*), a result similar to this study [29]. Other published MIC values for different kinds of honey against *C. albicans* lie between 18% and 42% (*v*/*v*) or >60% (*v*/*v*) in some studies [14,15,27,28,30].

Interestingly, the supplements in the MGH formulation have enhanced the antimicrobial activity of the honey formulation. Likely, this has been contributed by the antioxidants and vitamins C and E. Numerous clinical studies have shown that vitamins C and E can have synergistic effects with antibiotics and enhance the growth inhibition and the lethal effects of antibiotics against microorganisms [31,32,33,34,35,36,37,38,39,40]. Oliviera et al. investigated the antimicrobial activity of honey and L-Mesitran against *S. pseudintermedius* isolates and *M. pachydermatis* fungi [41]. In line with our study, L-Mesitran demonstrated enhanced antimicrobial activity compared to honey, which were likely contributed by the other components in the MGH formulation, such as the vitamins C and E [41]. Other supplements include lanolin and PEG4000. Lanolin is applied to treat dry and irritated skin, as it aids in restoring the epidermal barrier and reducing water loss of the skin [42]. Lanolin is sometimes said to be antimicrobial, but there is limited scientific literature to substantiate this. As an emollient, it penetrates the skin into intracellular spaces to form an emulsion with epidermal water which in turn can be released into the dry stratum corneum layer where *Candida* spp. adhere and multiply in the infected vaginal tissue [43,44,45,46] . These hydrating properties make it an excellent vehicle for retaining water-soluble antimicrobial agents, such as MGH [40,46]. Poly-ethylene-glycols (PEGs) are a family of water-soluble linear polymers. PEG4000 has a molecular weight of 4000 and is frequently used in a variety of pharmaceutical, cosmetic, ophthalmic applications and enhances drug delivery [47]. No direct antimicrobial activity has been described for PEG4000, however, it can improve the release of antimicrobial drugs [47].

The findings of this small-scale study offer insight into the fungicidal capacity of both pure honey and an MGH-based compound at pH values 4.0 and 4.6 and 5.2, which are typically seen in VVC as well as the healthy vagina [48]. The results of this study demonstrate some minor strain-specific antifungal variation in MICs at different pH effects, but a concentration of minimally 50% (*v*/*v*) L-Mesitran was demonstrated to have activity against all isolates, at all pH levels tested. Few of the reviewed studies that have investigated pure honey’s ability to inhibit *Candida* spp. investigated the effect of pH [49]. 

The contribution of honey in alleviating vaginal discomfort caused by *Candida* infections has been demonstrated in various in vivo studies where by day 8 fewer individuals suffered from symptoms including inflammation, vaginal discharge, itching, and dyspareunia in the honey group as compared to azole control groups [27,49,50]. The ointments evaluated in these studies consisted of either pure unprocessed honey or homogenous ointments with honey contents of 50–70% (*v*/*v*). The effect an MGH formulation would have when being delivered to the site of infection remains to be elucidated. Therefore, future research should investigate whether the deposition of an MGH formulation enriched by antioxidant supplements could convey anti-inflammatory and immunostimulatory properties on site to effectively control vaginal *Candida* infections. The gel evaluated in this study has been the subject of a small Dutch anecdotal clinical study. In their study, Boon (2002) evaluated immune cell infiltration, bacterial and yeast growth and vaginal complaints in thirty symptomatic women following the vaginal application of L-Mesitran [51]. Overall reactions of the women were positive and microscopy of the vaginal flora before and after the seven-day treatment period with the MGH ointment showed rapid eradication of yeast cells and reduction of inflammation. MGH forms a promising alternative to antifungal drugs without the risk of developing resistance. Since these findings imply the feasibility of the clinical vaginal application of MGH, future research should be conducted on the clinical application of MGH formulations whilst evaluating the treatment time, patient experience, and comfort. 

## 5. Conclusions

In conclusion, in vitro research should not disregard the potential effect of environmental pH on antimicrobial susceptibility of vaginal pathogens. The MGH formulation L-Mesitran has antifungal activity against clinical *Candida* albicans isolates at minimum 50% *v*/*v* dilution (a honey content of at least 20% *v*/*v*). The vitamins supplemented to L-Mesitran may enhanced the antimicrobial activity, since a concentration of 40% honey did not show antifungal activity. This data supports the further investigation of L-Mesitran in clinical studies as an alternative to available OTC fungistatic drugs in conditions such as VVC.

## Figures and Tables

**Table 1:** Summary of tested compounds, their composition, and test conditions.

Test compound and Composition	Concentrations Tested	Equivalent Honey Concentration	Tested pH Levels
Raw honey used in L-Mesitran ^1^	40%, 20%, 10%, 5%	40%, 20%, 10%, 5%	5.2, 4.6, 4.0
L-Mesitran soft wound gel ^2^	50%, 25%, 12.5%, 6.25%	20%, 10%, 5%, 2.5%	5.2, 4.6, 4.0

^1^ 100% pure honey; ^2^ 40% medical grade honey (MGH).

## Data Availability

The data that support the findings of this study are available from the corresponding author, E. van der Werf, upon reasonable request.
